# Single isocenter HyperArc treatment of multiple intracranial metastases: Targeting accuracy

**DOI:** 10.1002/acm2.14234

**Published:** 2023-12-07

**Authors:** Fang Li, Noor Mail, Maria Stefania diMayorca, Travis J. McCaw, Cihat Ozhasoglu, Ronald Lalonde, Jina Chang, Mohammed Saiful Huq

**Affiliations:** ^1^ Radiation Oncology UPMC Hillman Cancer Center Pittsburgh Pennsylvania USA; ^2^ Radiation Oncology Montefiore Medical Center Bronx New York USA; ^3^ Radiation Oncology University of Pittsburgh School of Medicine and UPMC Hillman Cancer Center Pittsburgh Pennsylvania USA

**Keywords:** HyperArc, intracranial SRS/SBRT

## Abstract

**Purpose/objectives:**

(**A)** To examine the alignment accuracy of CBCT guidance for brain metastases with off centered isocenters, (**B)** to test dose delivery and targeting accuracy for single isocenter treatments with multiple brain metastases. We report the results of the end‐to‐end test for Truebeam stereotactic radiosurgery (SRS).

**Materials/methods:**

An anthropomorphic CT head phantom was drilled with five MOSFET inserts and two PTW Pinpoint chamber inserts. The phantom was simulated, planned, and delivered. For the purpose of comparing the accuracy of alignment, CBCTs were acquired with the isocenter centered and offset superiorly 8 cm, inferiorly 8 cm, anteriorly 7 cm, posteriorly 7 cm, and right 5 cm. There were six degrees of freedom corrections applied to the plans, as well as intentional rotational and translational errors for dose comparisons. Dose accuracy checks were performed with MOSFET and PTW Pinpoint chamber, and targeting accuracy was assessed with GafChromic films.

**Result:**

(**A)** Compared to centered CBCT, off‐centered CBCT scan showed some alignment errors, with a maximum difference of 0.6‐degree pitch and 0.9 mm translation when the phantom was placed 8 cm inferior off center. **(B)** For the single isocenter plan, measured doses of the five MOSFET were 95%–100% of the planned dose, whereas the multiple isocenter plans were 96%–100%. With intentional setup errors of 1‐degree pitch, doses were 97.1%–100.4% compared to the perfect setup. The same was found for the two pinpoint chamber readings with 1‐degree rotation and 1 mm translation. (**C)** Targeting accuracy for targets at the isocenter is 0.67 mm, within the machine specification of 0.75 mm. Targeting accuracy for isocenters 6–12 cm away from the target is in the range 0.67–1.18 mm.

**Conclusion:**

(**A)** Single isocenter HyperArc treatments for multiple brain metastases are feasible and targeting accuracy is clinically acceptable. (**B)** The vertex in a cranial scan is very important for proper alignment.

## 1 INTRODUCTION

The introduction of HyperArc (HA) (Varian Medical System, Palo Alto, CA), an automated noncoplanar volumetric modulated arc therapy (VMAT) approach, has generated widespread discussion in the literature^1^, ^2^, ^3^, ^4^, ^5^, ^6^, ^7^, ^8^ on the topic of single‐isocenter stereotactic radiosurgery (SRS) and stereotactic radiotherapy (SRT) of multiple brain metastases. Treatments conducted with a single isocenter have the obvious advantage of reducing treatment time. Shortening the treatment time reduces the burden on clinical resources as well as the likelihood of motion within the fractions.[Supplementary-material acm214234-supitem-0001]

Ruggieri et al.^4^ compared HA with MultipleBrainMets (MBM) (BrainLab AG, Munchen, Germany), a single‐isocenter approach using dynamic conformal arcs, and found no significant difference in quality between the two approaches. HA plans had a slightly higher Paddick conformity index, but there were no significant differences in gradient index, mean target dose, mean brain dose, and brain V12 Gy. In addition, they showed sufficient agreement between planned and delivered dose distributions for each technique, with HA plans slightly outperforming MBM plans, on average.

It has been demonstrated that Gamma Knife (Elekta Instruments, Stockholm) and Cyberknife (CK) (Accuray Inc., Sunnyvale, CA) produce optimal plans in a review of SRS technology by Skourou et al.^5^ Its frame and intra‐fractional correction from Cyberknife also contribute to superior accuracy. The use of adaptive c‐arm linacs, however, demonstrated similar quality plans with the addition of efficiency and accessibility.

Calvo et al.^6^, ^7^ showed that dosimetrically comparable plans could be created regardless of whether the isocenter was in the target. When an IMRS plan with a single‐isocenter is designed, off‐isocenter targets within 6 cm of the linac isocenter can be targeted within 1 mm. Thus, an “off‐target” isocenter may be employed to avoid potential collisions during CBCT acquisition. This solution allows them to provide SRS treatments quickly, without the risk of collision, and with reduced time and effort when treating multiple targets simultaneously.

Although using a single isocenter treatment for multiple lesions has many benefits, targeting accuracy remains an issue. Yoon et al.^9^ have demonstrated that plans with shallower dose gradients have improved target coverage when targets are misaligned, particularly for targets smaller than 1 cm. By considering the increasing 12‐Gy volume, they argue that shallower dose gradients can be used for frameless brain SRS/SRT procedures to reduce coverage deficiency caused by targeting errors. In a recent study by Kraft et al.,^10^ multiple metastases were successfully treated with a single‐isocenter VMAT technique with high local control rates. These control rates were robust to increase targets' distance from the isocenter, supporting the concept of using a single isocenter to treat several brain metastases at once.

We have been treating multiple brain metastases with TrueBeam LINAC with a high‐definition multi‐leaf collimator (MLC) (2.5 mm leaf width in the center, 5 mm leaf width off‐center starting +/– 4 cm from the central axis) and multiple isocenter approaches. For these treatments, we utilize only the central 2.5 mm‐wide MLC leaves. In our experience, many CBCT scans were acquired with the imaging centered at the treatment isocenter, which was off‐center of the skull. One of our concerns was the accuracy of the alignment for these peripherally centered scans. Another major concern for the single‐isocenter technique is the accuracy of the targeting since small targets that are located far from the isocenter would be more susceptible to rotational setup errors. The purpose of this study was to assess the accuracy of CBCT alignment for off‐centered CBCT, determine the accuracy of end‐to‐end targeting for single isocenter treatments, and determine the appropriateness of the 1 mm margin between PTV and GTV.

## 2 MATERIALS AND METHODS

### 2.1 CT simulation and treatment planning

An anthropomorphic CK head phantom with a removable ball cube in the middle of the brain of the Lucy Phantom (Standard Imaging, USA—Wisconsin) was drilled to have five MOSFET inserts (TN‐502RD‐H, Best Medical, Ontario, Canada) and two Pinpoint chamber inserts (PTW 31016, Freiburg, Germany). With each hole being 2–3 cm deep, the sensitive volume of the detector is placed inside the brain, avoiding shallow buildup regions. An insert conforming to the detector was inserted into the hole, followed by the pouring of melted wax. A CT image was acquired in order to verify that there were no gaps around the detectors. The phantom was then simulated using a GE LightSpeed 64 Slice CT Scanner with a slice thickness of 1.25 mm. An isocenter was established in the middle of the brain and three‐point localization marks were drawn on the mask.

**FIGURE 1:**
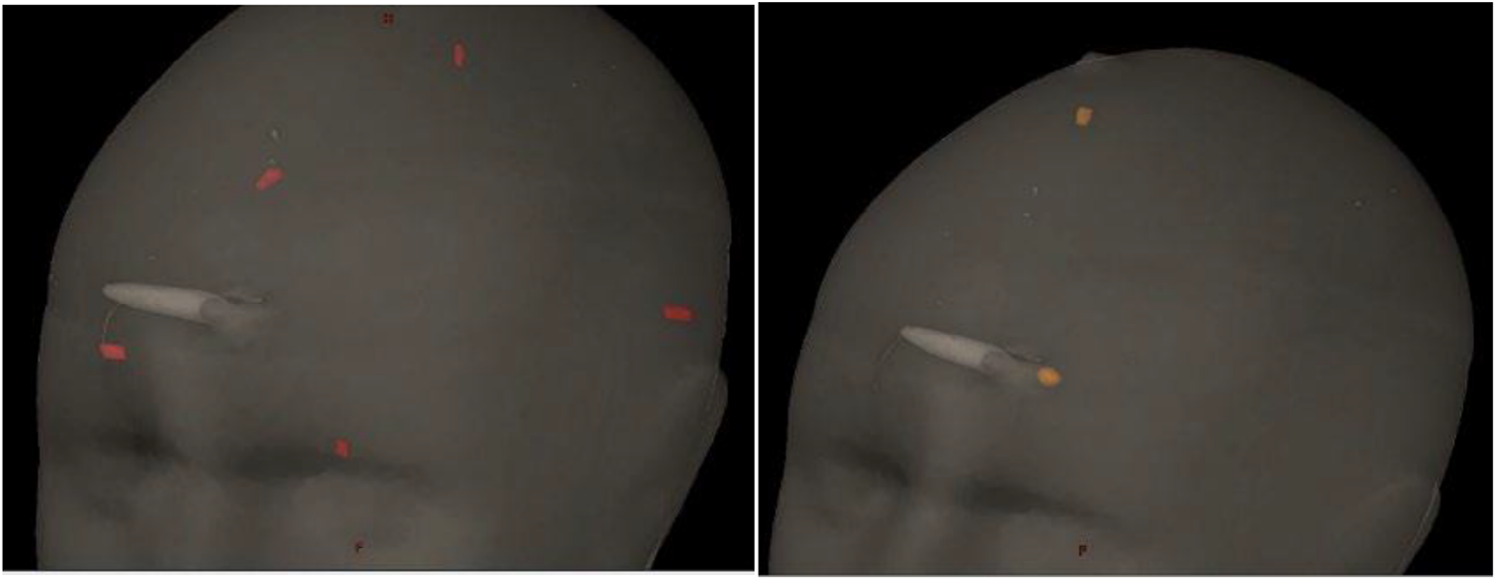
Five MOSFET (red at Left image) and two Pinpoint (yellow at right image) contours.

The phantom CT was imported into the Eclipse treatment planning system (version 15.6, Varian, Palo Alto, US). There were five MOSFETs contoured as GTV1‐5 (each with a volume of 0.02 cc), and there were two pinpoint chambers contoured as GTV6‐7 (each with a volume of 0.02 cc), as shown in Figure 1. A GTV contour was drawn only for the active detector volume. A margin of 1 mm was added between the GTV and the PTV. In addition, the ball was contoured as GTV8, and then orthogonal pairs of GafChromic film were inserted into its center to evaluate targeting accuracy. The HA plans were then generated using 6 MV flattening‐filter‐free (6FFF) photon beams at a dose rate of 1400 MU/min. In the case of single brain mets (BMs), the isocenter is placed at the center‐of‐mass of the brain metastasis. When there are multiple BMs, the default isocenter for HA was placed at the geometrical center between all BMs. One full arc is used in HA planning with a couch rotation of 0°, and three partial noncoplanar arcs are used with couch rotations of 315°, 45°, and 90°. A calculation grid of 1 mm was used as part of the AAA 15604 algorithm.

For absolute dosimetry, we had three plans (A, B, C) for the MOSFETs and one plan (D) for the pinpoint chamber:

Plan A: Targeting MOSFETs 1,2,5 – PTV 1,2,5 (using thin MLC leaves), with isocenters of 3, 6.4, and 7 cm from PTV1, PTV2, and PTV5; Plan B: Targeting MOSFET 3,4 – PTV 3,4 (use thin MLC leaves), isocenter is 5 and 4 cm away from PTV3 and PTV4, respectively; Plan C: Targeting MOSFET 1−5 (use all MLC leaves, 1 iso), isocenters are located 4.8, 7.5, 5.9, 8.1, and 7.8 cm from PTV 1–PTV 5 respectively. Plan D: Targeting two pinpoint chambers—PTV 6,7 (use all MLC leaves, one isocenter for both); the isocenters are located 5.5 and 5 cm from PTV 6 and PTV 7, respectively.

For the purpose of catching treatment errors, all contours are limited within the active volume of the detector, and the HA plans have been designed to be uniform and conformal. Plan A–D have a conformity index of 1.17, 1.22, 1.22, and 1.21, respectively; gradient measures are 0.69, 0.67, 0.79, and 0.68 cm, respectively; and maximum doses are 10.89, 10.83, 10.87, and 10.75 Gy, respectively.

Using a ball cube as a target, we created four plans to test the targeting accuracy of GTV8 with end‐to‐end test: “P0” isocenter is in the center of the target, “P6” isocenter is 6 cm away, “P9” is 9 cm away, and “P12” is 12 cm away.

### 2.2 CBCT alignment

Before the experiment, IsoCal calibration^11^ was conducted to calibrate the CBCT and machine radiation isocenters. Using the QFix mask and support, the head phantom was positioned on the 6D couch. Prepositioning the head phantom was achieved using an infrared optical positioning system. A standard head full fan protocol was used for the acquisition of all CBCT data (100 kVp, 150 mAs, 25 cm^2^ field of view, *G* = 20 to 180E) and the reconstruction of all slices was performed with a slice thickness of 1 mm. A CBCT‐to‐“planning CT” registration was then performed using online auto‐matching, including the entire skull, and setting the intensity range to bone. Following the online 6D alignment, corrections were sent to the treatment couch and the shifts were recorded.

In order to test the positioning accuracy of off‐center CBCT scans, the couch was moved to set the phantom off‐center by a known distance: SUP 8, IN 8, ANT 7, POST 7, Rt 5 cm, and so forth. It mimics the clinical scenario when targets are very peripheral, and so the patient's head will not be centered. A comparison was made between the shifts from the off‐center scans and those from the centered CBCT scans. Before any off‐center movement, we centered the couch as the home position; the home position was the baseline. The accuracy of the couch shift was confirmed by performing a verification CBCT.

We repeated the above two tests after replacing the ball cube with wax sheets in the phantom. By doing so, we aimed to create a phantom that looked more like an actual patient, without obvious high contrast structures in the middle of the brain, since the ball cube contains high contrast and six embedded fiducials that would artificially improve alignment accuracy.

### 2.3 Treatment delivery for absolute dosimetry

The MOSFET and pinpoint chamber detectors were calibrated to record the absolute dose. The ADCL calibration factor used for pinpoint chambers 1 and 2 were 2.51152 and 2.52418 Gy/nC. Winston‐Lutz^12^ tests were conducted prior to each delivery. The lasers were used to position the phantom. After a centered‐CBCT alignment and online 6D corrections, the Perfect‐Pitch couch applied the shifts with 0.1 mm precision, and the plans were delivered. On different days, for five days, we recorded dose measurements for plans A to D.

We intentionally moved the couch with a rotation of 1 degree and a translation of 1 mm and compared the dose measurements with those recorded with the perfect setup after delivering the plans with perfect alignment. For targets up to 12 cm off‐isocenter, a 0.5‐degree rotation error would require a 1 mm margin; similarly, for targets up to 6 cm off‐isocenter, a 1‐degree rotation error would require a 1 mm margin. Our practice of 1 mm GTV to PTV margin was tested dosimetrically to determine if it is sufficient for residual setup errors.

### 2.4 Targeting accuracy by end‐to‐end test

Using pre‐cut orthogonal GafChromic EBT‐3 films (Prototek California LLC, San Jose, CA), we inserted two films into the preexisting slits of the Ball Cube. The plans P0‐12 were delivered with perfect 6D alignment, with 0.5 degree and 1 degree rotation errors, respectively.

With an EPSON scanner (48‐bit Gray, 300 dpi, *W* = 2.75, *H* = 7.81), irradiated films were scanned pairwise with a blank film serving as a background, and the targeting accuracy was assessed with E2E Film Analysis software. As part of the E2E analysis, the software compares the centroid of the actual dose distribution on the exposed film with the centroid of the planned distribution.

Moreover, we delivered the off‐center plans (P0‐P12) intentionally rotated by 0.5–1° to determine the resulting targeting error.

## 3 RESULTS

### 3.1 CBCT alignment

#### 3.1.1 Lucy with Ball Cube

For off‐centered CBCTs, compared to centered CBCTs (without skull cutoff), the maximum couch shift difference is 0.7 degrees rotation with negligible translational differences.

#### 3.1.2 Lucy without Ball Cube

A greater difference was observed between the calculated shifts and those calculated without the Ball Cube (Table 1), with a maximum shift difference of 0.6 degrees pitch and 0.9 mm vrt. The largest shift difference was observed when the phantom was placed 8 cm inferior to the center.

**TABLE 1:** Shift difference for head phantom without Ball Cube.

Couch shift	Center #1	Rt 5 cm #2	Sup 8 cm #3	Inf 8 cm #4	Rt 5 cm Sup 8 cm #5	Rt 5 cm Inf 8 cm #6	Post 7 cm #7	Ant 7 cm #8
**Vrt (cm)**	0	0.07	0	−0.9	0.04	−0.9	0.01	0.01
**Lng (cm)**	0	−0.02	−0.06	0.06	−0.1	0.04	−0.04	0.03
**Lat (cm)**	0	−0.01	−0.04	0.03	−0.03	0.04	0.08	−0.04
**Rotation (deg)**	0	−0.01	0.1	0.2	0.3	0.2	0	0.1
**Pitch (deg**	0	0	0.4	0.6	0.3	0.5	−0.1	0
**Roll (deg)**	0	0.02	0.1	0.1	0.3	0.1	−0.2	0.2

### 3.2 Dosimetry

Each plan was delivered on five different days and the results were averaged.

Initially, we delivered the plan with a perfect 6D alignment; as shown in Table 2, the measured doses are in agreement with the planned dose.

**TABLE 2:** Planned versus measured dose for Plan A–D with perfect alignment.

	A PTV 1,2,5 Dose (Gy)			B PTV 3,4 Dose (Gy)	
	GTV1	GTV2	GTV5	GTV3	GTV4
**Planned dose (Gy)**	10.35	10.27	10.51	11.38	9.32
	(10.27–10.55)	(10.13–10.51)	(10.11–10.81)	(10.86–11.77)	(8.97–11.43)
**Mean measured dose (Gy)**	10.1	10.1	10.5	11.1	10.3
**Dose ratio**	97.60%	98.50%	99.90%	96.00%	96.00%
**SD**	0.1	0.3	0	0.1	0.2
	**C PTV1–5 Dose (Gy)**				
	**GTV1**	**GTV2**	**GTV3**	**GTV4**	**GTV5**
**Planned dose (Gy)**	10.69	10.61	10.42	9.73	10.68
	(10.5–10.9)	(10.3–10.7)	(9.8–10.6)	(0.1–10.1)	(9.9–10.9)
**Mean measured dose (Gy)**	10.66	10.48	9.86	9.55	10.4
**Dose ratio**	99.70%	98.80%	94.60%	98.20%	97.40%
**SD**	0.08	0.04	0.05	0.19	0.11
	**D PTV 6,7 Dose (Gy)**				
	**GTV6**	**GTV7**			
**Planned dose (Gy)**	10.11 (10.19–10.39)	10.22 (10.16–10.31)			
**Mean measured dose (Gy)**	9.65	9.78			
**Dose ratio**	95.50%	96%			
**SD**	0.02	0.03			

We then delivered Plans C and D with some intentional setup errors and compared them to the perfect 6D alignment delivered the same day. The results of MOSFET Plan C and pinpoint chamber Plan D are presented in Table 3.

**TABLE 3:** Measured dose for Plan C–D with and without perfect alignment.

	C PTV1–5 Dose (Gy)				
	PTV1	PTV2	PTV3	PTV4	PTV5
**Perfect 6D alignment**	10.2	9.84	10.5	9.71	10.9
**1 deg pitch**	10.1	9.64	10.2	9.75	10.6
**Dose ratio**	99.0%	98.0%	97.1%	100.4%	97.2%

	**D PTV6, 7 Dose (Gy)**	
	**PTV6**	**PTV7**
**Perfect 6D alignment**	9.65	9.78
**1 deg pitch**	9.55	9.68
**Dose difference**	99.0%	99.0%
**1 deg roll**	9.57	9.75
**Dose difference**	99.2%	99.7%
**1 deg rotation**	9.64	9.75
**Dose difference**	99.9%	99.7%
**1 mm Vrt**	9.66	9.78
**Dose difference**	100.2%	100.0%
**1 mm Lng**	9.58	9.85
**Dose difference**	99.3%	100.7%
**1 mm Left**	9.56	9.82
**Dose difference**	99.1%	100.4%

### 3.3 Targeting accuracy

**FIGURE 2:**
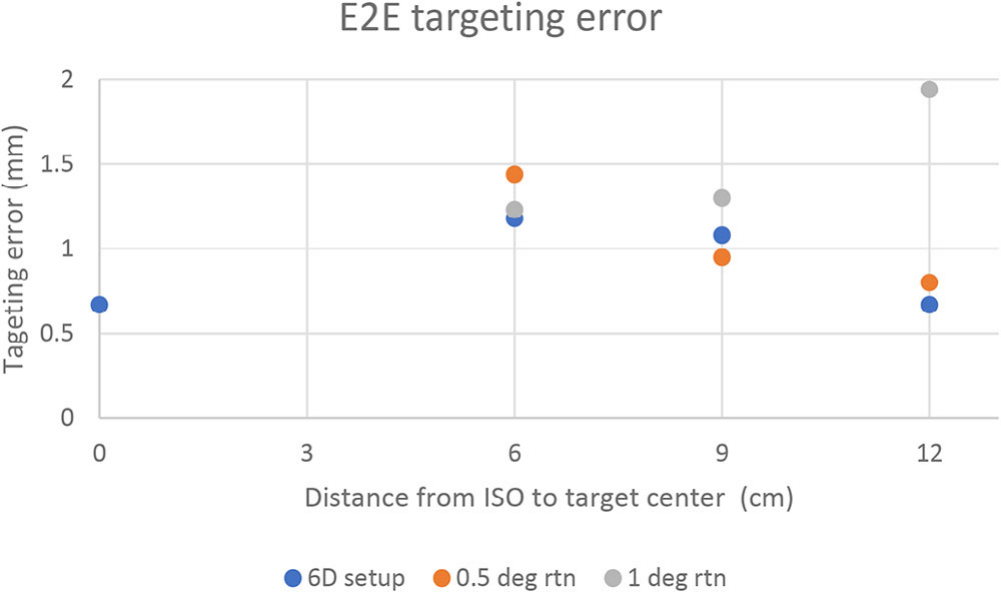
E2E targeting accuracy for iso 0−12 cm away from the center of target.

The E2E results from GafChromic films are shown in Figure 2.

The targeting accuracy was 0.67 mm when the isocenter was positioned in the center of the target (Figure 2). It is consistent with our daily Winston‐Lutz results, which show a mean delta of 0.39 mm and a maximum total delta of 0.59 mm. When the isocenter was placed 6–12 cm away from the target center, accuracy ranged between 0.67 and 1.18 mm (the blue dot); with a setup error of 0.5 degrees, accuracy ranged from 0.8 to 1.44 mm (the red dot); and with a 1‐degree rotational setup error, accuracy ranged from 1.23 to 1.94 mm (the grey dot).

**FIGURE 3:**
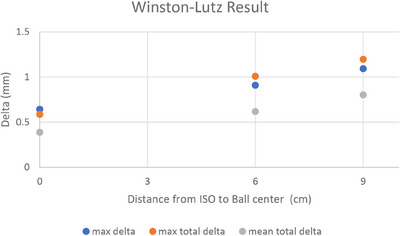
Winston‐Lutz for iso 0−9 cm away from the center of target.

As the distance between the isocenter and the target increased from 6 to 12 cm, targeting accuracy or conformity is slightly improved. It might be attribute to a chance as this is only a single measurement at each iso‐to‐target center distance without uncertainty analysis. Clearly, this unexpectedly improved target accuracy is not significant, it is lying within the system uncertainty from Winston‐Lutz test^22^ data (Figure 3) for the cube phantom at the Isocenter (0.69 ± 0.38 mm) and off‐Isocenter position (1.01 ± 0.19 mm). The targeting accuracy for 0 and 0.5 degrees was almost constant as a function of the distance between the isocenter and the target. As a result of the intended 1‐degree couch rotation, the targeting error for the P12 plan was 1.94 mm, which was in good agreement with the expected error of 2 mm. The exact reason remains a puzzle for us for further studies.

## 4 DISCUSSION

The present study investigated the impact of the location of the isocenter on the alignment and overall targeting accuracy of the TrueBeam SRS system. As per the SRS protocol at our center, the expansion of 1 mm from GTV to PTV is primarily the setup errors. As a result, the alignment accuracy of cranial brain metastases on TrueBeam must be submillimeter. In this study, the positioning accuracy of CBCT for TrueBeam SRS was evaluated for various isocenter locations using an anthropomorphic head phantom. An IsoCal calibration was performed prior to the tests, and a Winston‐Lutz test was performed using a cube phantom in order to verify the accuracy of the CBCT system. The offset between the IsoCal‐calibrated image centers and the Winston‐Lutz radiation isocenter was found to be within 0.4 mm in a study conducted by Du et al.^13^ A Winston‐Lutz test offset of 0.75 mm was observed in our center daily. In scanning the head phantom with the ball cube insert, which contains six embedded fiducials, where the high contrast aids in CBCT alignment, the misalignment from off‐centered scans was only 0.7 degrees, with almost no translation errors. In real patients, CBCT and CT image registration is primarily based on rigid skull alignment, therefore a wax cube was substituted for a ball cube. After replacing the ball cube with wax, the off‐centered CBCT misalignment increased to 0.9 mm and 0.6 degrees.

A specific finding of the study was that cranial alignment can be improved by ensuring that the patient's head position and the couch are centered, especially if the target is located near the base of the skull. If the head is not centered and the vertex is cut off, we observed misalignments of up to 0.9 mm and 0.6 degrees. In addition to Graulieres,^14^ Kim et al.^15^ also found an isocenter variation of approximately 1 mm for CBCT. It is in accordance with the findings of Huang et al.^16^ and Li et al.^17^

In addition, the accuracy of off‐isocenter target positioning was assessed, with the majority of the deviations being less than 1 mm. According to Ezzel et al.,^18^ CBCT‐based alignment was within 1.1 mm for distances between the isocenter and target of 10 cm or more. Similarly, Kim et al.^14^ found that the alignment of off‐isocenter targets was less than 1 mm on a Novalis Tx Linac.

This slight misalignment between the CBCT and radiation isocenters is not significant for small setup errors and single target treatments. However, CBCT and machine coordinate alignment should be carefully considered in order to treat multiple metastases with a single isocenter, in addition to the alignment of the isocenter as determined by IsoCal or WL. The deviation between imaging and treatment coordinates is greater at greater distances from the isocenter. According to the study by Tominaga et al.,^19^ off‐isocenter target positioning with a Varian Novalis TX is less accurate than target positioning at the isocenter.

When the head phantom was aligned without high‐contrast objects in the brain, it exhibited an off‐centered CBCT misalignment of up to 0.9 mm and 0.6 degrees. If we prepositioned a patient with ISO marks for very peripheral lesions such as those of the cerebellum, the superior and anterior parts of the skull would not be visible on CBCT. This will have a negative effect on the alignment. Instead of prepositioning the CBCT based on patient iso marks, we should perform it with the isocenter in the middle of the skull and then shift it afterward. Second, if the isocenter is in the center of the target, the targeting accuracy is acceptable with the 1 mm GTV to PTV margin. In accordance with Graulieres,^14^ who reported an increase in target positioning accuracy from 0.69 mm at the isocenter to 1.3 mm as a function of distance from the isocenter, this finding is also supported by our findings.

There is a limitation to this E2E test; the centroid of the 70% isodose line must be located in the center of the ball. Our targeting accuracy test is adapted from Cyberknife, in which the Cyberknife plan goes through several iterations to align the target isodose line within 0.03 mm of the target area of interest. There is no such function in Eclipse. Accordingly, we optimized the Eclipse plan in such a way that the 70% isodose line encompasses the ball and the centroid of the 70% isodose sphere is within the centroid of the ball. To compare the centroid location of the 70% isodose line with the center of the ball, we converted the 70% isodose line into a structure. When the two coordinates differed in any direction, we had to re‐optimize until the two coordinates were perfectly aligned.

According to our findings, a 1 mm margin of GTV to PTV is appropriate when the isocenter is located in the center of a target. This is in agreement with the data presented by Agazaryan^20^ and Nakan et al.o^21^ To achieve targeting accuracy, a greater GTV to PTV margin is required when planning multiple targets and using a single isocenter. In our current practice, we perform MV imaging at every couch angle and limit the residual setup error to 0.5 mm and 1 degree. This has been found to be appropriate. As the targeting error or accuracy is within the 1 mm margin of GTV to PTV expansion, we limit the residual setup error to within 0.5 mm and 0.5 degrees.

## 5 CONCLUSION

In the case of multiple intracranial targets, targeting accuracy can be acceptable for a single isocenter HA SRS/SRT if the farthest targets are within 12 cm of the isocenter. In order to minimize alignment errors, care should be taken to center the skull for CBCT acquisition and to register the entire skull, especially the vertex.

## AUTHOR CONTRIBUTIONS

*Data collection*: Fang Li, Noor Mail, Maria diMayorca, and Jina Chang. *Discussion about the ideas and details of method*: Travis Mccaw, Cihat Ozhasoglu, Ron Lalonde, and Saiful Huq. *Statistical analysis*: Fang Li. All authors edited the manuscripts.

## CONFLICT OF INTEREST STATEMENT

The authors declare no conflicts of interest.

## Supporting information

Supporting Information

## References

[1] Parikh NR , Kundu P , Levin‐Epstein R , et al. Time‐driven activity‐based costing comparison of stereotactic radiosurgery to multiple brain lesions utilizing single‐isocenter vs. multiple‐isocenter technique. Int J Radiat Oncol Biol Phys. 2019;105(1):E84‐E85. doi:10.1016/j.ijrobp.2020.06.035 32603774

[2] Hughes RT , Masters AH , McTyre ER , et al. Initial SRS for patients with 5 to 15 brain metastases: results of a multi‐institutional experience. Int J Radiat Oncol Biol Phys. 2019;104(5):1091‐1098. doi:10.1016/j.ijrobp.2019.03.052 30959122

[3] Cullom ET , Xia Y , Chuang KC , et al. Single isocenter SRS using CAVMAT offers improved robustness to commissioning and treatment delivery uncertainty compared to VMAT. J Appl Clin Med Phys. 2021;22(7):36‐43. 10.1002/acm2.13248 PMC8292691 34165217

[4] Ruggieri R , Naccarato S , Mazzola R , et al. Linac‐based radiosurgery for multiple brain metastases: comparison between two mono‐isocenter techniques with multiple non‐coplanar arcs. Radiother Oncol. 2019;132:70‐78. 30825972 10.1016/j.radonc.2018.11.014

[5] Skourou C , Hickey D , Rock L , et al. Treatment of multiple intracranial metastases in radiation oncology: a contemporary review of available technologies. BJR Open. 2021;3:20210035. 34877458 10.1259/bjro.20210035 PMC8611687

[6] Ho HW , Yang CC , Lin HM , et al. The new SRS/FSRT technique HyperArc for benign brain lesions: a dosimetric analysis. Sci Rep. 2021;11(1):1‐11. 34702859 10.1038/s41598-021-00381-9 PMC8548509

[7] Calvo‐Ortega JF , Moragues S , Pozo M , Delgado D , Casals J . Dosimetric feasibility of an “off‐target isocenter” technique for cranial intensity‐modulated radiosurgery. Med Dosim. 2015;40(4):279‐284. 25824421 10.1016/j.meddos.2015.02.003

[8] Calvo‐Ortega JF , Pozo M , Moragues S , Casals J . Targeting accuracy of single‐isocenter intensity‐modulated radiosurgery for multiple lesions. Med Dosim. 2017;42(2):104‐110. 28478867 10.1016/j.meddos.2017.01.006

[9] Yoon JW , Park S , Cheong KH , Kang SK , Han TJ . Combined effect of dose gradient and rotational error on prescribed dose coverage for single isocenter multiple brain metastases in frameless stereotactic radiotherapy. Radiat Oncol. 2021;16(1):1‐8. 34465331 10.1186/s13014-021-01893-4 PMC8406565

[10] Kraft J , van Timmeren JE , Mayinger M , et al. Distance to isocenter is not associated with an increased risk for local failure in LINAC‐based single‐isocenter SRS or SRT for multiple brain metastases. Radiother Oncol. 2021;159:168‐175. 33798610 10.1016/j.radonc.2021.03.022

[11] Komiyama R , Ohira S , Ueda H , et al. Intra‐fractional patient motion when using the Qfix Encompass immobilization system during HyperArc treatment of patients with brain metastases. J Appl Clin Med Phys. 2021;22(3):254‐260. 33656261 10.1002/acm2.13143 PMC7984469

[12] Chiu TD , Yan Y , Foster R , Mao W . Long‐term evaluation and cross‐checking of two geometric calibrations of kV and MV imaging systems for Linacs. J Appl Clin Med Phys. 2015;16:306‐310. 10.1120/jacmp.v16i4.5140 PMC5690018 26218992

[13] Du W , Johnson JL , Jiang W , Kudchadker RJ . On the selection of gantry and collimator angles for isocenter localization using Winston‐Lutz tests. J Appl Clin Med Phys. 2016;17:167‐178. 26894350 10.1120/jacmp.v17i1.5792 PMC5690203

[14] Graulieres E , Kubler S , Martin E , Ferrand R . Positioning accuracy of a single‐isocenter multiple targets SRS treatment: a comparison between Varian TrueBeam CBCT and BrainLab ExacTrac. Physica Medica. 2020;80:267‐273. 33221708 10.1016/j.ejmp.2020.10.022

[15] Kim J , Wen N , Jin JY , et al. Clinical commissioning, and use of the Novalis Tx linear accelerator for SRS and SBRT. J Appl Clin Med Phys. 2012;13(3):3729. 10.1120/JACMP.v13i3.3729 22584170 PMC5716565

[16] Huang Y , Zhao B , Chetty IJ , Brown S , Gordon J , Wen N . Targeting accuracy of image‐guided radiosurgery for intracranial lesions: a comparison across multiple linear accelerator platforms. Technol Cancer Res Treat. 2016;15(2):243‐248. 25759427 10.1177/1533034615574385

[17] Li F , Park J , Lalonde R , et al. Is Halcyon feasible for single thoracic or lumbar vertebral segment SBRT? J Appl Clin Med Phys. 2021;23(1):e13458. 34845817 10.1002/acm2.13458 PMC8803290

[18] Ezzell GA . The spatial accuracy of two frameless, linear accelerator‐based systems for single‐isocenter, multitarget cranial radiosurgery. J Appl Clin Med Phys. 2017;18(2):37‐43. 10.1002/acm2.12044 PMC5689957 28300379

[19] Tominaga H , Araki F , Shimohigashi Y , et al. Accuracy of positioning and irradiation targeting for multiple targets in intracranial image‐guided radiation therapy: a phantom study. Phys Med Biol. 2014;59(24):7753. 25419723 10.1088/0031-9155/59/24/7753

[20] Agazaryan N , Tenn S , Lee C , et al. Simultaneous radiosurgery for multiple brain metastases: technical overview of the UCLA experience. Radiat Oncol. 2021;16(1):1‐9. 34789300 10.1186/s13014-021-01944-w PMC8597274

[21] Nakano Hisashi , Tanabe S , Sasamoto R , et al. Radiobiological evaluation considering setup error on single‐isocenter irradiation in stereotactic radiosurgery. J Appl Clin Med Phys. 2021;22(7):266‐275. 34151498 10.1002/acm2.13322 PMC8292684

[22] Eagle Anton , Tallhamer M , Keener J , Geneser S . A simplified and effective off‐axis Winston–Lutz for single‐isocenter multi‐target SRS. J Appl Clin Med Phys. 2023;24(2):e13816. 36420972 10.1002/acm2.13816 PMC9924106

