# Supplementary material for: Single isocenter HyperArc treatment of multiple intracranial metastases: Targeting accuracy
Source: J Appl Clin Med Phys. 2023 Dec 7;25(1):e14234. doi: 10.1002/acm2.14234 (PMC10795440; doi:10.1002/acm2.14234)
Supplement: Supplementary file 1 — Supporting Information [file ACM2-25-e14234-s001.docx]

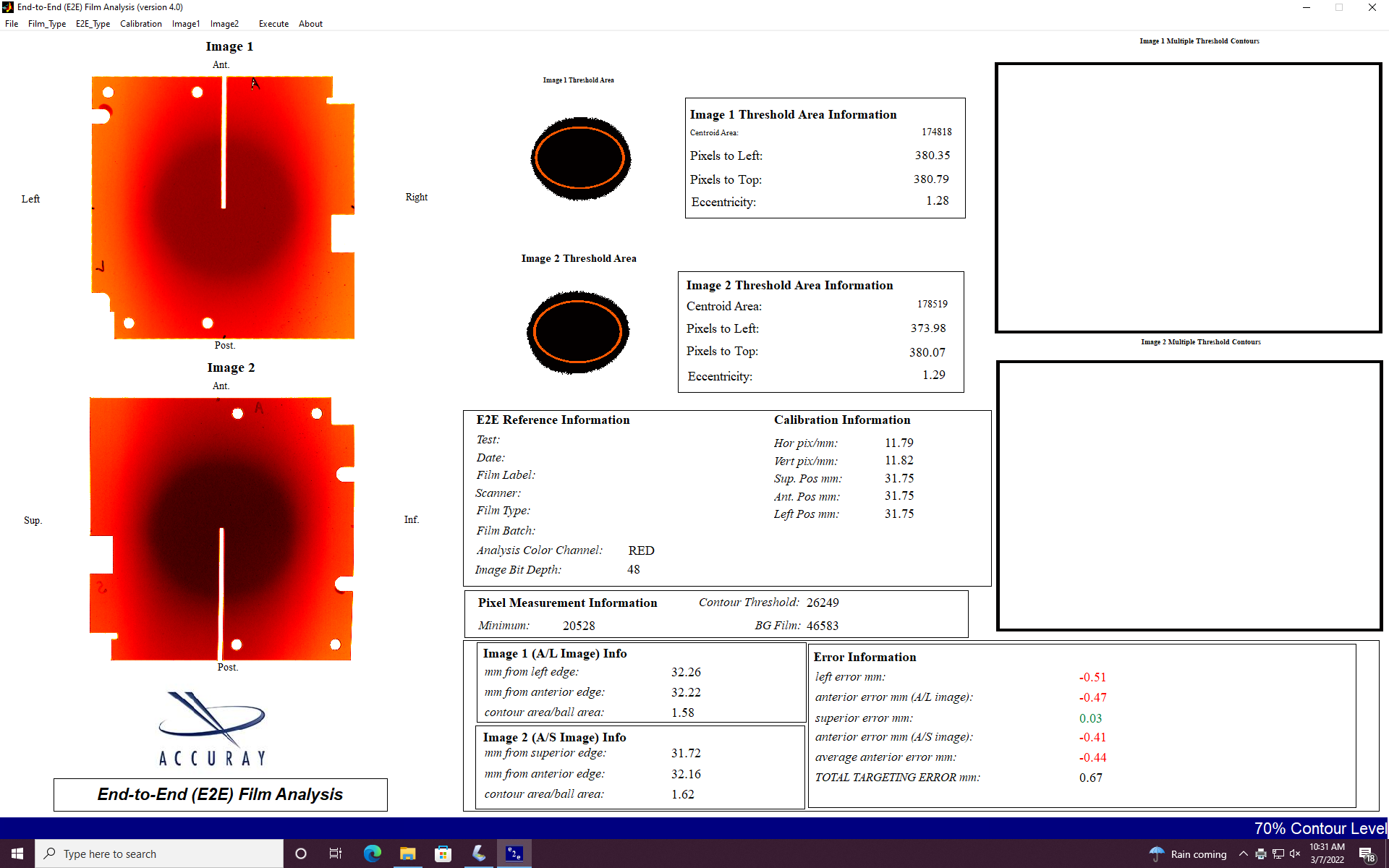


*Fig.1 6D setup with Isocenter at the target center*

*
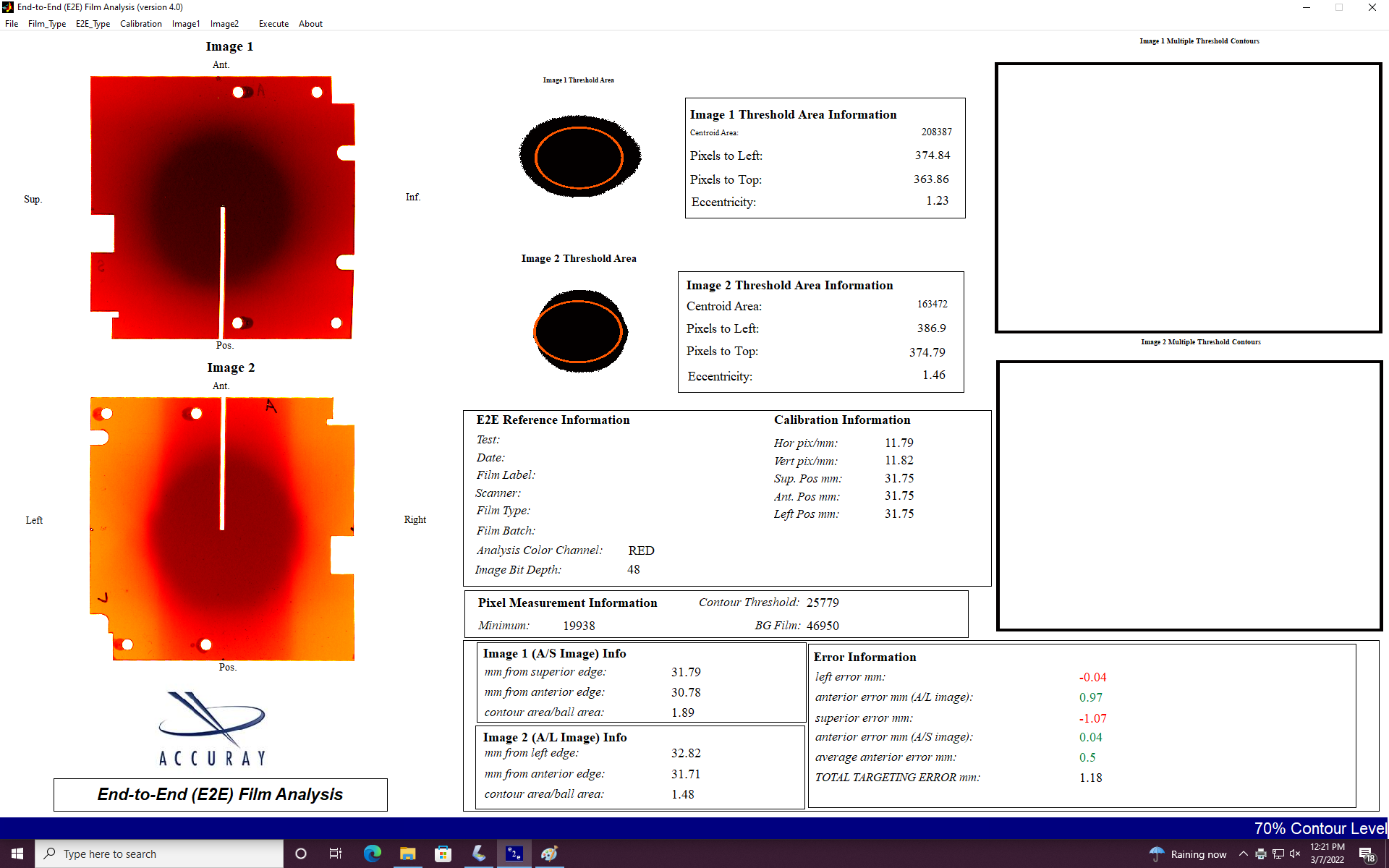
*

*Fig.2 6D setup with Isocenter 6cm from the target center*

*
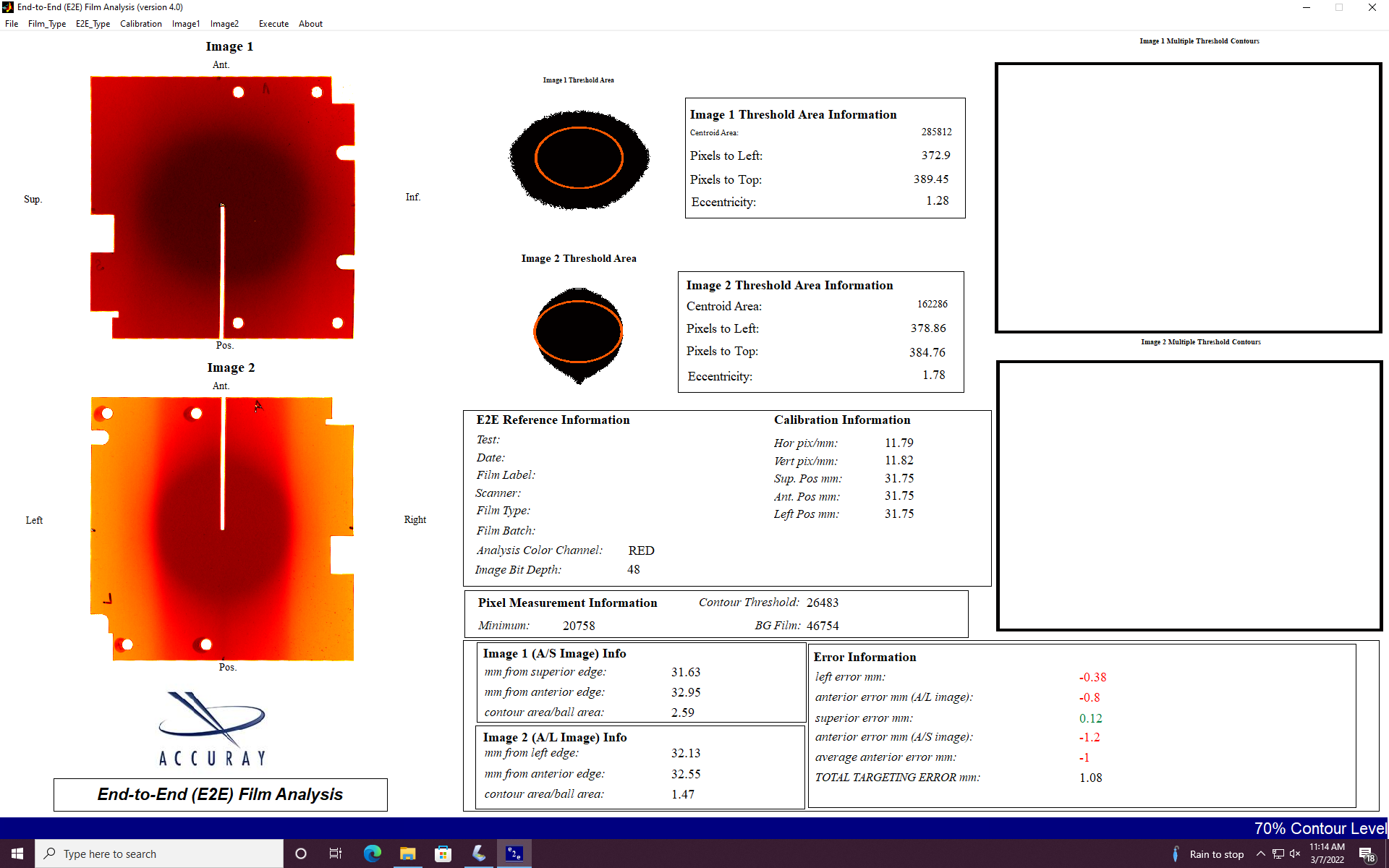
*

*Fig.3 6D setup with Isocenter 9cm from the target center*

*
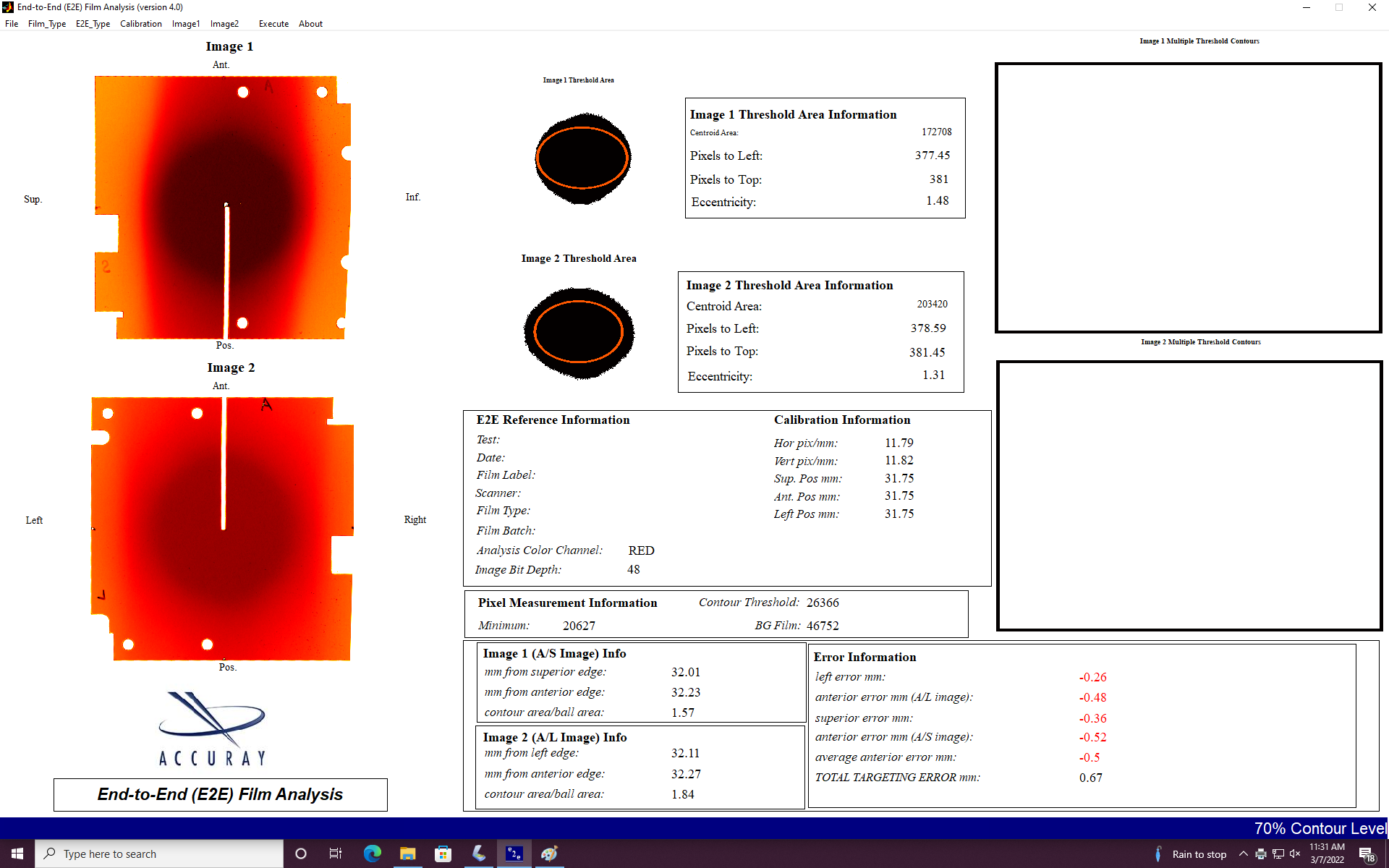
*

*Fig.4 6D setup with Isocenter 12cm from the target center*

*
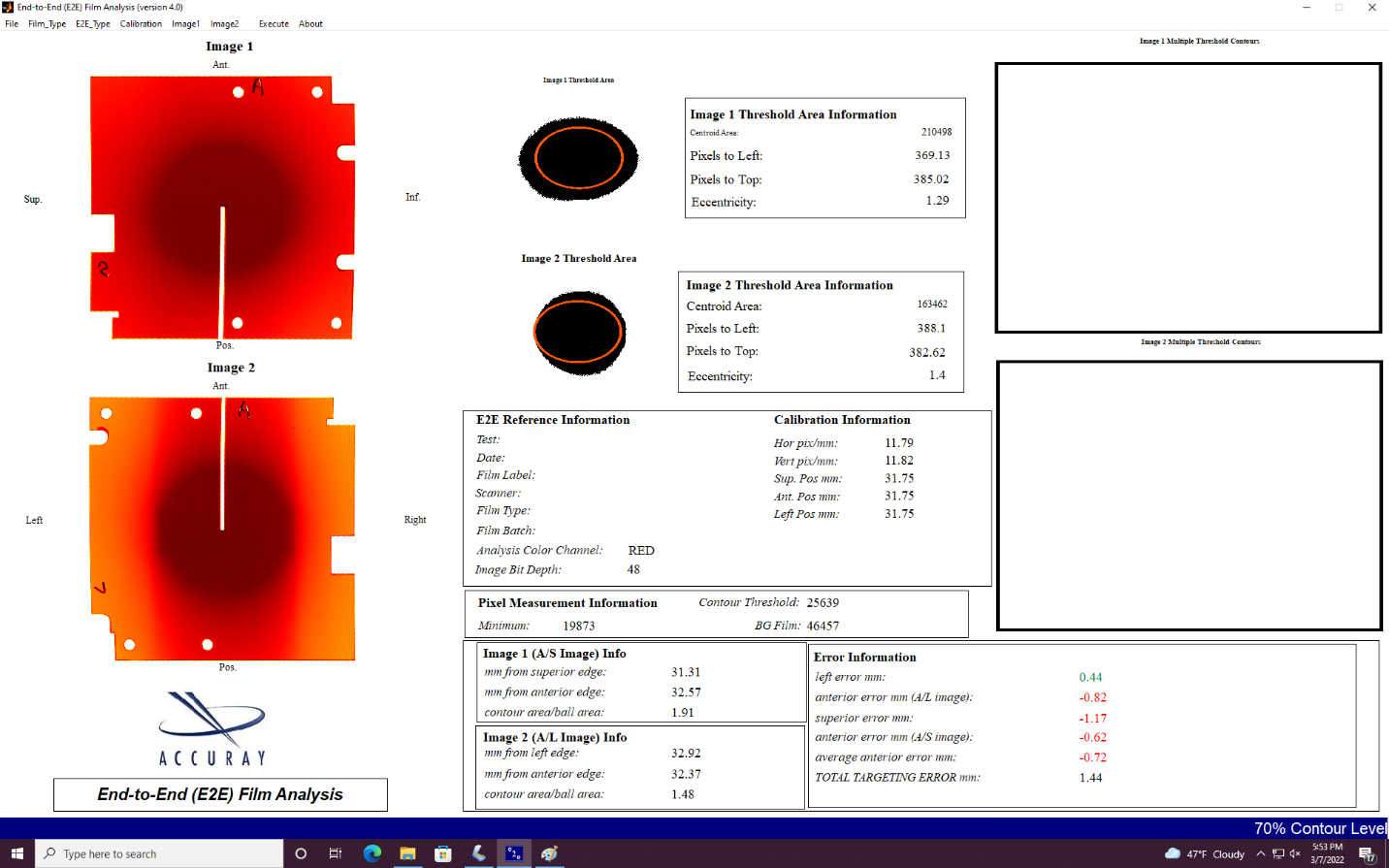
*

*Fig.5 0.5° residual rotation with Isocenter 6cm from the target center*

*
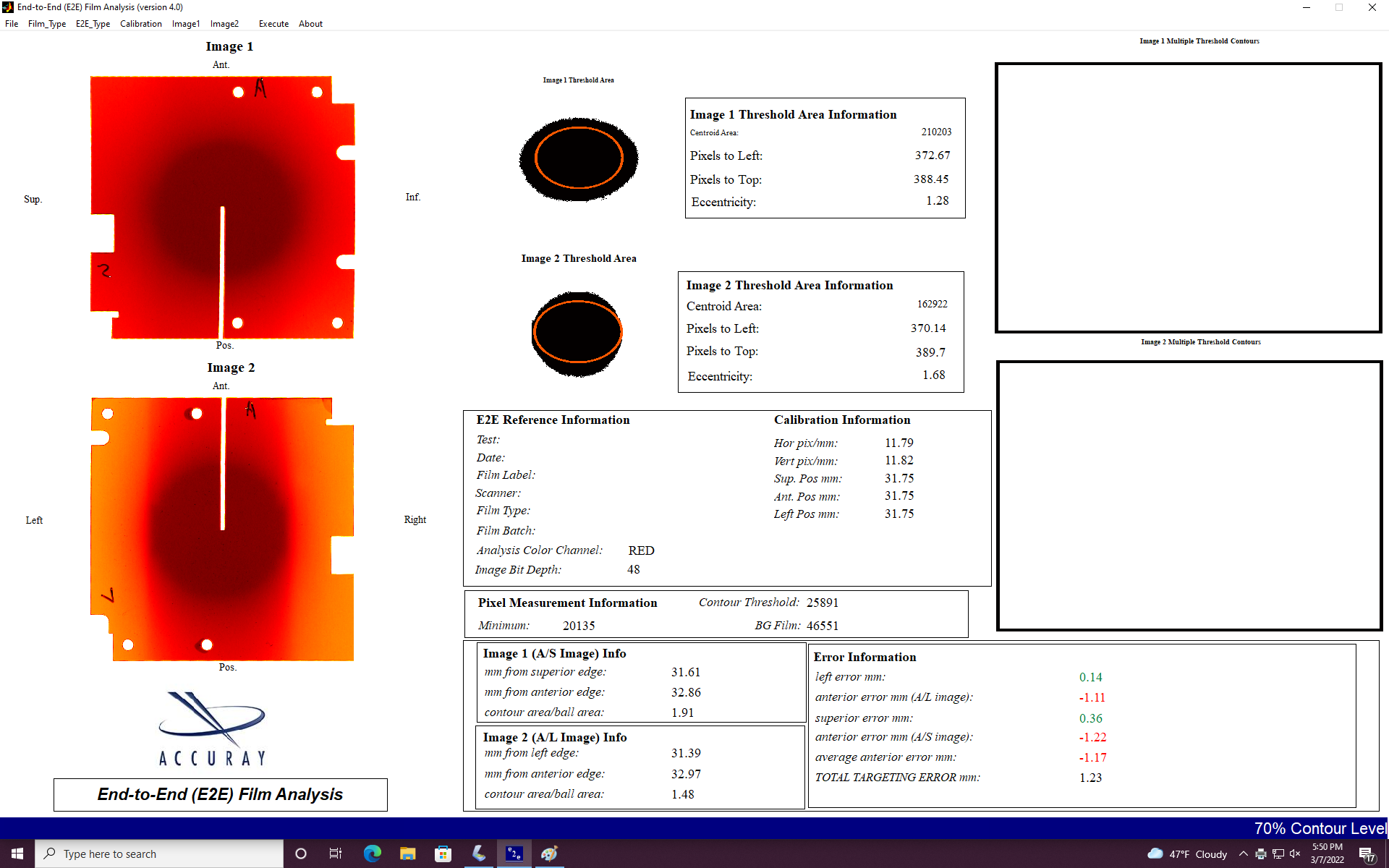
*

*Fig.6 1° residual rotation with Isocenter 6cm from the target center*

*
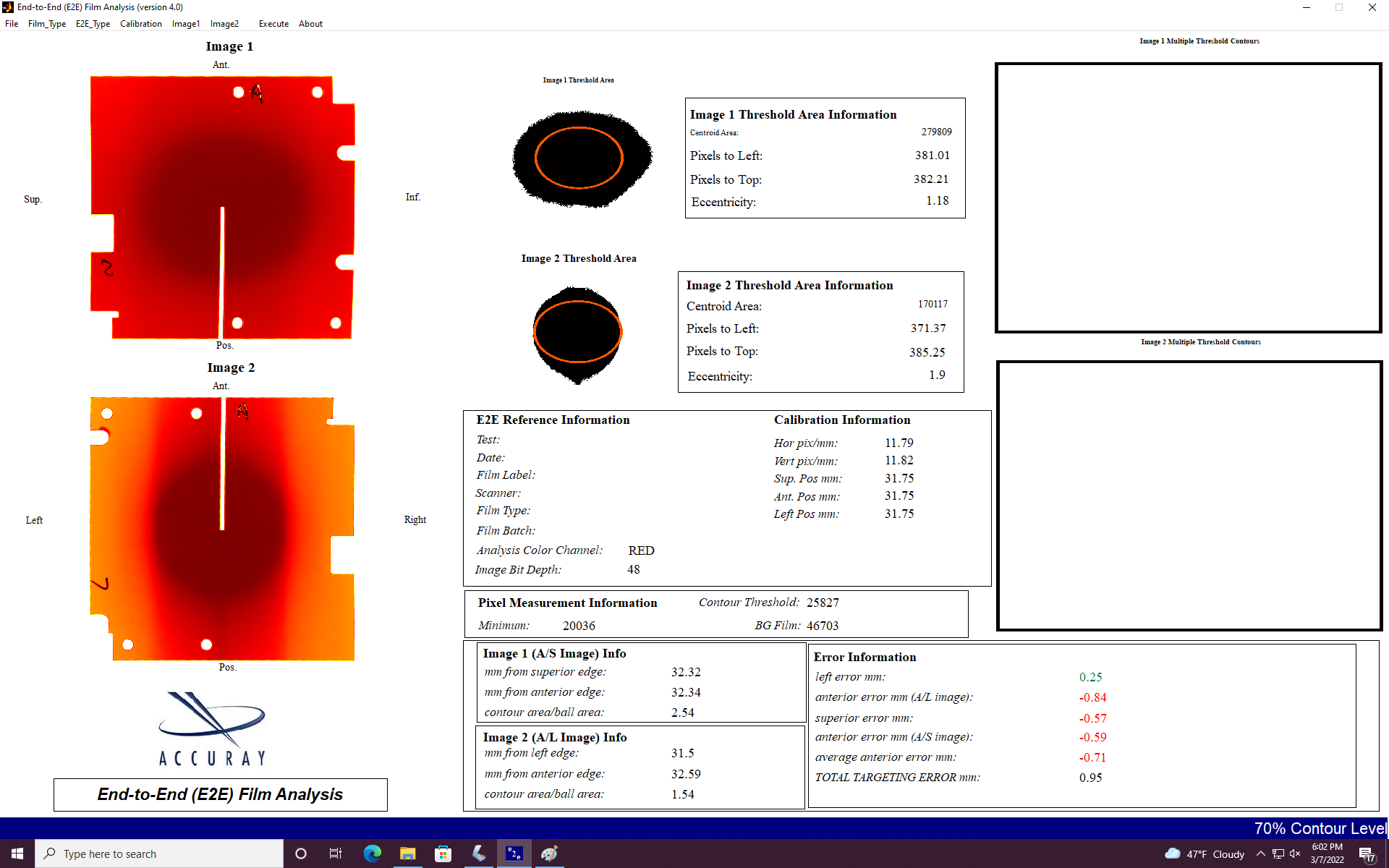
*

*Fig.7 0.5° residual rotation with Isocenter 9cm from the target center*

*
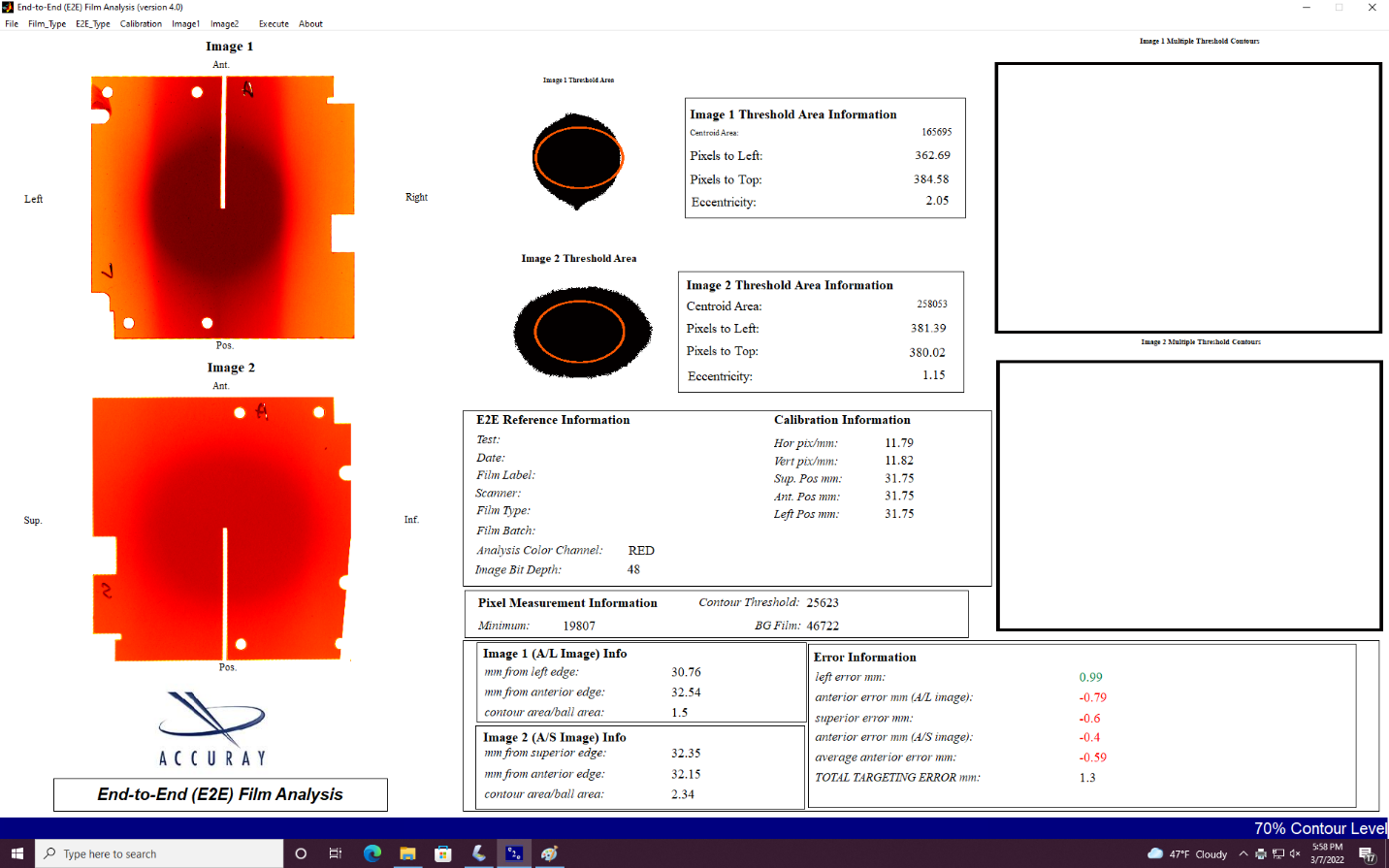
*

*Fig.8 1° residual rotation with Isocenter 9cm from the target center*

*
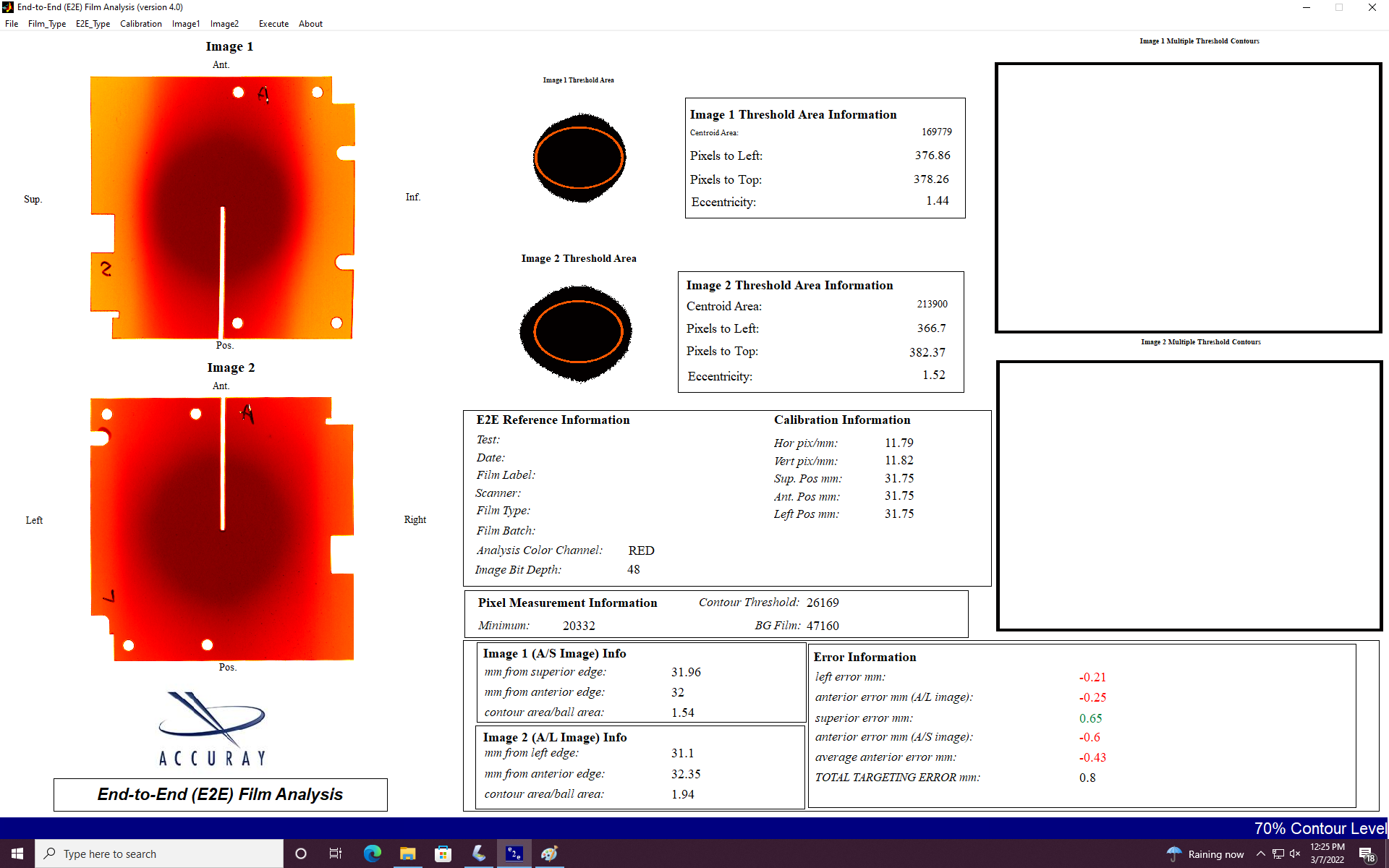
*

*Fig.9 0.5° residual rotation with Isocenter 12cm from the target center*

*
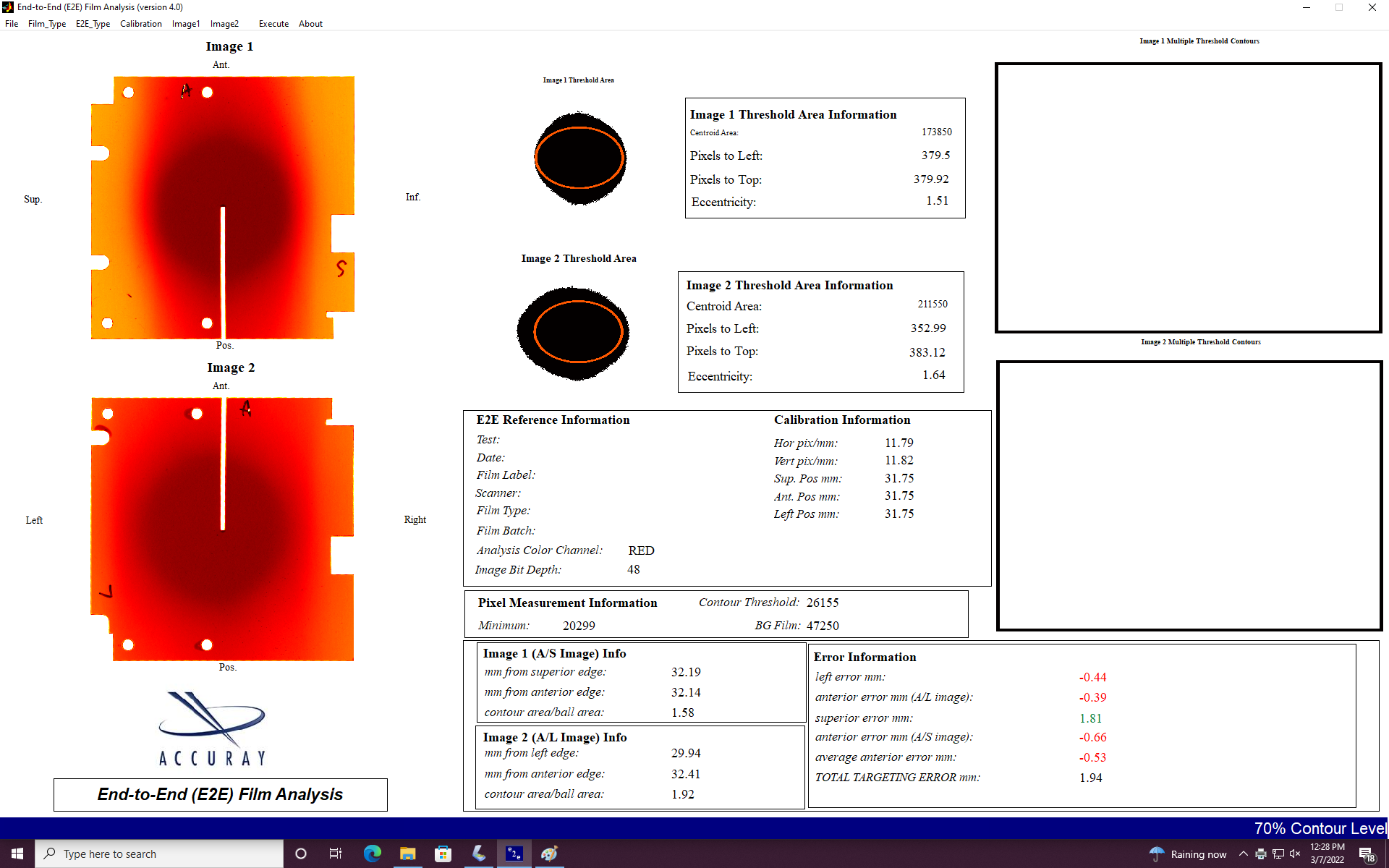
*

*Fig.10 1° residual rotation with Isocenter 12cm from the target center*

***Table 1. Margin for targets away from isocenter***

| **iso to target distance r (mm)** | **patient rotation θ (°)** | **safety margin (mm)** |
| --- | --- | --- |
| 60 | 0.5 | 0.5 |
| 70 | 0.5 | 0.6 |
| 80 | 0.5 | 0.7 |
| 90 | 0.5 | 0.8 |
| 100 | 0.5 | 0.9 |
| 110 | 0.5 | 1.0 |
| 120 | 0.5 | 1.0 |
| 60 | 1 | 1.0 |
| 70 | 1 | 1.2 |
| 80 | 1 | 1.4 |
| 90 | 1 | 1.6 |
| 100 | 1 | 1.7 |
| 110 | 1 | 1.9 |
| 120 | 1 | 2.1 |

***Table 2. off center Winston-Lutz***

|  |  |  | **10/2** | **10/4** | **10/6** | **10/9** | **10/11** |
| --- | --- | --- | --- | --- | --- | --- | --- |
|  | **3D** | **3D x L/R** | -0.1 | 0.25 | 0.45 | 0.8 | -0.42 |
|  |  | **3D y S/I** | 0.1 | -0.33 | 0.5 | -0.32 | 0.43 |
| **WL 0** |  | **3D z A/P** | -0.16 | -0.09 | 0.38 | -0.38 | 0.28 |
|  | **delta** | **max delta** | 0.42 | -0.63 | 0.77 | -0.98 | -0.42 |
|  |  | **max total delta** | 0.5 | 0.09 | 0.84 | 1.08 | 0.43 |
|  |  | **mean total delta** | 0.25 | -0.09 | 0.66 | 0.67 | 0.28 |
|  |  |  |  |  |  |  |  |
|  |  |  | **10/2** | **10/4** | **10/6** | **10/9** | **10/11** |
|  | **3D** | **3D x L/R** | 0.44 | 0.52 | 0.83 | 0.95 | 0.41 |
|  |  | **3D y S/I** | 0.25 | -0.54 | -0.26 | -0.05 | -0.34 |
|  |  | **3D z A/P** | -0.07 | -0.58 | -0.43 | -0.38 | -0.69 |
| **WL 6** | **delta** | **max delta** | 0.62 | -0.93 | -1.03 | 1.13 | 0.84 |
|  |  | **max total delta** | 0.72 | 1 | 1.19 | 1.17 | 0.96 |
|  |  | **mean total delta** | 0.47 | 0.74 | 0.66 | 0.62 | 0.6 |
|  |  |  |  |  |  |  |  |
|  |  |  | **10/2** | **10/4** | **10/6** | **10/9** | **10/11** |
|  | **3D** | **3D x L/R** | 0.6 | 0.65 | 0.95 | 0.97 | 0.55 |
|  |  | **3D y S/I** | -1.08 | 0.51 | -0.47 | -0.35 | -0.39 |
|  |  | **3D z A/P** | -0.15 | -0.38 | -0.25 | 0.73 | -0.52 |
| **WL 9** | **delta** | **max delta** | -1.45 | 1 | -0.94 | -1.17 | -0.9 |
|  |  | **max total delta** | 1.6 | 1.06 | 0.99 | 1.33 | 1.01 |
|  |  | **mean total delta** | 1.18 | 0.76 | 0.72 | 0.73 | 0.63 |
